# Assessing the spatial distribution and sources of heavy metal pollution in the snow cover: A case study from Pavlodar, Northeastern Kazakhstan

**DOI:** 10.1371/journal.pone.0322300

**Published:** 2025-05-12

**Authors:** Alina Faurat, Dinara Yessimova, Gulmira Satybaldiyeva, Askhat Kuatbayev, Aizhan Utarbayeva, Ainagul Kaliyeva, Kairat Akhmetov, Shujaul Mulk Khan, Zeeshan Ahmad, Seilkhan Rakhmanov

**Affiliations:** 1 Department of Geography and Tourism, Toraighyrov University, Pavlodar, Kazakhstan; 2 Department of Ecology, Saken Seifullin Kazakh Agrotechnical University, Astana, Kazakhstan; 3 Department of Biology and Ecology, Toraighyrov University, Pavlodar, Kazakhstan; 4 Department of Plant Sciences, Quaid-i-Azam University Islamabad, Islamabad, Pakistan; 5 CAS Key Laboratory of Tropical Forest Ecology, Xishuangbanna Tropical Botanical Garden, Chinese Academy of Sciences, Mengla, Yunnan, China; Bangladesh Council of Scientific and Industrial Research, BANGLADESH

## Abstract

This study assesses heavy metal contamination in the snow cover of northeastern Kazakhstan by analyzing both the melted filtrated water and the solid sediment after filtration near various pollution sources. The research examines the impact of oil refining, thermal power plants (northern industrial zone), aluminum production (eastern industrial zone), and transportation on heavy metal dispersion. Results indicate that Zn, Cr, and Pb concentrations in the solid phase of snow in residential areas exceed those in industrial zones, reaching 436.6, 259.1, and 218.6 mg/kg, respectively. The highest overall concentrations were found for barium (949.4 mg/kg) and manganese (638.1 mg/kg). In the liquid fraction (meltwater), Zn (58.6 μg/l) and Sr (34.8 μg/l) were predominant, while Mn (28.3 μg/l) was the main pollutant in the eastern industrial zone. Dust load values in the snow cover ranged from 42.3 to 418.5 mg/m²/day, with the highest pollution load observed for Cd, Pb, and Mo. Despite variations in dust load across the city (135.5 mg/m²/day in the northern industrial zone, 152.3 mg/m²/day in the eastern industrial zone, and 147.1 mg/m²/day in residential areas), the overall dust pollution level remains low. However, a sanitary-hygienic assessment revealed that most heavy metal concentrations in snow exceed maximum permissible levels for soil in areas influenced by industrial facilities and transportation, except for Mo, V, and Mn. The ecological risk index of snow pollution in Pavlodar was calculated at 192.13, indicating a high potential ecological risk. These findings highlight the importance of snow as an indicator of environmental pollution and the need for continuous monitoring to assess urban contamination trends.

## Introduction

Sustainable urban development is increasingly recognized as a critical component in addressing the environmental challenges associated with urbanization. As cities grow, they face significant pressures related to environmental degradation, including air and water pollutions, loss of biodiversity and increased greenhouse gas emissions. The interaction between urbanization and environmental quality is complex and requires a multifaceted approach to urban planning and environmental policy development [1].

In addition, urbanization cause significant changes in land use that can exacerbate environmental degradation. Studies show that urban expansion can degrade environmentally sensitive areas, especially if urban growth is not effectively managed [2].

Sustainable urban development emphasizes the integration of economic, social and environmental dimensions, suggesting that well-planned expansion can improve environmental quality. However, it is crucial to consider the spatial and temporal dynamics of pollution, as localized issues also have a wider regional impacts due to spatial spillovers [3].

Heavy metal pollution in industrial cities is a serious environmental problem with widespread impacts on both ecosystems and human health. Industrial activities, including mining, metallurgy and manufacturing, have been identified as significant sources of heavy metal pollution in various regions, resulting in widespread contamination of soil, water and food sources [4–11]. The main sources of heavy metal and metalloid pollution are both natural phenomena such as rock weathering, mineralisation, dust storms and volcanic eruptions, and various industrial processes [12]. Industrial activities contribute to heavy metal emissions through processes such as fossil fuel combustion (emitting As, Cu, Co, Cr, V, Ni, Sb, Fe, Mn, Zn, Sn), oil combustion (Mn, Pb, Fe, Ni), vehicle exhaust (Pb, Cu, Cr, Sn, Sb), metal smelting (Ni, Cu, As, Pb, Cd), iron and steel production (Cr, Mn, Ni, Co), waste incineration (Pb, Zn) and cement production [13]. Numerous studies have assessed the pollution levels and environmental risks associated with heavy metals in different industrial cities [6,8–10]. Studies in Zhejiang, China, show that about 70% of the area is prone to heavy metal pollution, especially nickel, chromium and zinc [7], which is also found in Lanzhou, highlighting the long-term nature of this environmental problem [14].

The presence of heavy metals in urban dust is a major environmental concern due to its potential impact, directly, on human health and ecosystem integrity. Numbers of studies have investigated the levels, sources and associated risks of heavy metal pollution in urban dust in various cities around the world. These investigations have used a variety of analytical techniques to quantify heavy metal concentrations and assess the potential health risks associated with exposure to these pollutants [15–18]. Research in Lublin, Poland [18] has shown that heavy metals from street dust are leached by precipitation and accumulation in aquatic sediments. Estimating heavy metals in urban dust is a multifaceted challenge that requires comprehensive research to understand the sources, distribution patterns, and potential health effects posed by such pollutants [7,19].

Self-purification processes are continuously occurring in the atmosphere, mainly through two modes of trace element deposition: wet deposition (via precipitation) and dry deposition (as dust). Recent studies also show that dry deposition has a more significant effect on atmospheric purification due to its continuous and ubiquitous nature [7].

Snow includes both wet and dry precipitation and therefore acts as a deposition medium. Snow cover not only traps dust but also helps to clean the atmosphere when solid precipitation falls. The chemical composition of the snowpack (filtrate) is formed as a result of the deposition of various chemical elements from the atmosphere, the settling of solid particles and the absorption of water-soluble aerosols. When studying the snow cover, a two-phase analysis is carried out to determine the concentration of microelements in the solid phase (solid sediment after filtering) and the liquid phase of the snow (meltwater) [20]. Studies have shown that snow is a stable medium for monitoring atmospheric heavy metal pollution [21,22]. It can also serve as an indicator of pollution pathways [23–26]. Such research is of great relevance to the industrial city of Pavlodar in north-eastern Kazakhstan. The Pavlodar region, with the city of Pavlodar as its regional centre, is recognised as the largest industrial center in the country [27,28].

Studies in Pavlodar have highlighted the environmental impacts of industry, particularly heavy metal pollution and PM2.5 and PM10 emissions, which require further investigation [29,30]. Although studies of heavy metal content in the snow cover of Pavlodar were conducted in 2001–2002 [31,32], modern studies are important to compare data 20 years later.

In addition, the studied region is one of the industrial zone and major contributor in emissions of the air pollutants [33,34], as well as in environmental diseases among adults and children [35,36].

During the accumulation and melting processes, snow becomes a source of pollutants entering the soil and water bodies, leading to their secondary contamination (Vijayan et al., 2024) [37].

Previous studies confirm that transportation is the primary source of heavy metal pollution in urban snow cover, including zinc (Zn), copper (Cu), lead (Pb), cadmium (Cd), and nickel (Ni), as well as less-studied elements such as antimony (Sb), tungsten (W), and platinum group elements (PGEs) (Vijayan et al., 2024) [37]. An important factor exacerbating the pollution problem is global warming, which enhances the mobility and bioavailability of heavy metals in natural ecosystems. Changes in the hydrological regime, accelerated melting of snow and glaciers, and alterations in precipitation acidity can lead to intensified leaching of metals into soils and water bodies, increasing their environmental risk (Xiao et al., 2024) [38].

Additionally, recent studies on snow dumping sites have shown that they represent significant sources of environmental pollution. As snow accumulates in urban areas, it intensively absorbs microplastics and tire wear particles (TWP), which act as carriers of heavy metals and can significantly increase their concentration in meltwater. A study by Chand et al. (2024) [39] in Riga confirmed that heavy metal concentrations in urban snow dumping sites exceed background levels by an order of magnitude, and the leaching of these metals during snowmelt can have a severe impact on aquatic ecosystems.

Thus, the present study aims to assess the spatial distribution and sources of heavy metal pollution in the snow cover of Pavlodar, taking into account the latest research in this field. Unlike previous studies focusing on snow cover in other urbanized areas, this research provides the first comprehensive analysis of heavy metal content in the snow cover of a city with a highly developed industrial sector and significant transport load. The obtained results will not only help evaluate the extent of pollution but also contribute to the development of recommendations for reducing anthropogenic impact on the snow cover and the environment.

It was hypothesized that the concentrations of heavy metals in the snow cover vary according to the sources of pollution and the geographical location. The use of snow cover as a pollution indicator can provide an accurate assessment of the state of the environment and public health.

Therefore, the current study was aimed to assess the heavy metal contents in solid and liquid components of snow for determination of various pollution sources such as oil refineries, thermal power plants, Aluminium producing plants and transport. The study also aims to investigate the spatial distribution of pollution and dust loads and its impact on the sanitary condition of the urban environment.

In order to understand in detail the influence of anthropogenic sources on the pollution of snow cover in urban areas, this study addresses the following research questions

What are the concentrations of heavy metals in the solid and liquid fractions of the snowpack, and what types of pollution are most characteristic of different urban areas (industrial, residential)?

How do the dust load and the indicators of general pollution of the snow cover change according to the spatial distribution of the pollution and the degree of anthropogenic impact, and how does this affect the sanitary-and-hygienic condition of the urban environment?

Thus, the aim of the study is to assess the level and nature of heavy metal pollution of the snow cover in the conditions of an industrial city, to identify patterns of spatial distribution of pollutants and their potential impact on the environment and the sanitary and hygienic condition of the urban area.

In order to achieve the goal, the objectives of our research were set as follows to assess the content of heavy metals in the solid and liquid phases of the snow cover in different zones of Pavlodar city (1); to analyse the dust load and pollution indices of the snow cover, to determine the level of accumulation of pollutants (2); to determine the spatial distribution of pollution, to determine its characteristics depending on the functional zoning of the city (industrial, residential areas) (3); to carry out a sanitary and hygienic assessment of the pollution of the snow cover, comparing the heavy metal content with the maximum permissible concentrations and environmental standards (4).

We are of the opinion that this study can be used as a baseline study for countries and cities where snow falls to assess the particulate matter content of snow and to reconsider snow as a potential object of pollution monitoring.

## Materials and methods

### Study area

The study was conducted in Pavlodar, a city located in the northeastern part of Kazakhstan, within Pavlodar Region, which covers an area of 124.8 thousand km².

#### Physical and geographical characteristics of Pavlodar.

The city is situated in the middle reaches of the Irtysh River basin and is located on the first accumulative floodplain terrace of the river. This terrace gradually transitions into a lacustrine-eluvial denudation plain in the eastern part of the city. The topography is characterized by a gradual decrease in elevation from north to east, with absolute elevations ranging from 105 to 158 meters. The Irtysh River serves as the primary source of water supply for the city. Groundwater in the floodplain terraces lies at a depth of 1–17 meters, typically 2–5 meters. In the southeastern part of the basin, fresh water is widespread; however, as water-bearing rocks subside, the mineralization of groundwater increases.

#### Soil cover.

The region’s soil cover is represented by chestnut soils, which are classified as light loamy in texture. These soils form under dry steppe conditions and have a humus horizon (A + B) thickness of 40–50 cm, with a humus content of 3.5–4.0%. The soil cover is widely interspersed with solonetzic and solonchak soils, necessitating monitoring of their condition in the context of industrial impact.

#### Climate.

Pavlodar’s climate is characterized by sharp continentality and aridity, influenced by predominant anticyclonic air circulation, particularly in winter. The average temperature in January ranges from -17 to -19°C, while in July, it ranges from +20 to + 22°C. The annual precipitation varies between 220 and 300 mm, with 60–75% of precipitation occurring in the warm period (May–August), and the highest amounts typically recorded in June–July. During winter, significant soil freezing is observed, with depths reaching 87–162 cm [40].

#### Meteorological conditions and air pollution during the snow accumulation period in Pavlodar.

The period from October 2022 to January 2023 in Pavlodar was characterized by moderate to strong winds (7–14 m/s, with gusts reaching 22–23 m/s), with occasional fog and haze. Daily average temperatures ranged from +25°C in October to -34°C in December, and precipitation occurred in the form of rain and snow (0.0–10.4 mm).

Air quality monitoring indicated an elevated level of pollution during the winter period, particularly for suspended particles PM2.5 (up to 2.12 MPCmax), PM10 (up to 1.5 MPCmax), carbon monoxide (up to 2.12 MPCmax), and hydrogen sulfide (up to 2.51 MPCmax). The highest number of exceedances of the maximum permissible concentrations was recorded for PM2.5 (24 cases), PM10 (19 cases), carbon monoxide (102 cases), and hydrogen sulfide (88 cases). The impact of unfavorable meteorological conditions (UMC) on pollutant concentrations during this period was not observed [33].

Meteorological conditions during sampling: the weather in Pavlodar on January 30, from 9 p.m. on January 29–9 p.m. on January 30, 2023, was characterized by variable cloudiness with no precipitation. The wind was southwest at 5–10 m/s, and the air temperature in the afternoon ranged from -9 to -11°C.

**Fig 1:**
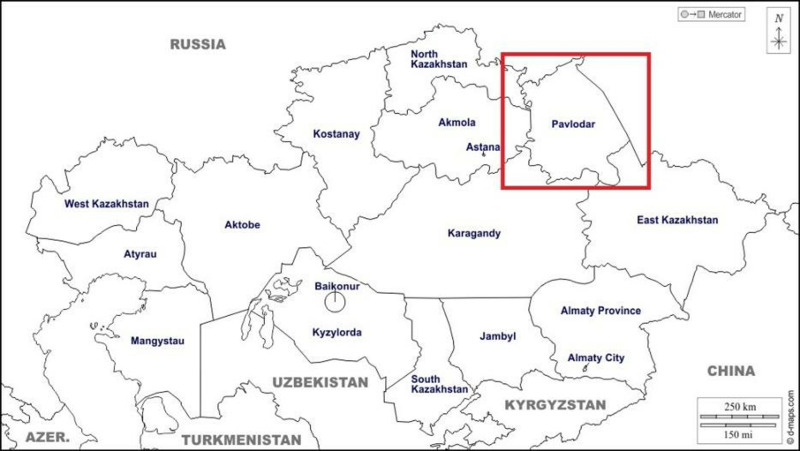
Map of the study area in Pavlodar, Kazakhstan [42]. Image source: Retrieved from https://d-maps.com/carte.php?num_car=15319&lang=ru.

The city of Pavlodar is one of the main industrial centres of the Republic of Kazakhstan (Fig 1). The predominant sector of local industry is metallurgy, which accounts for 42.6% of industrial output, followed by electricity with 17.8% and petroleum products with 13.3%. The city also has significant activity in the mechanical engineering and chemical industries, according to the Decree of the Government of the Republic of Kazakhstan of 12 June 2018 (No. 337).

**Fig 2:**
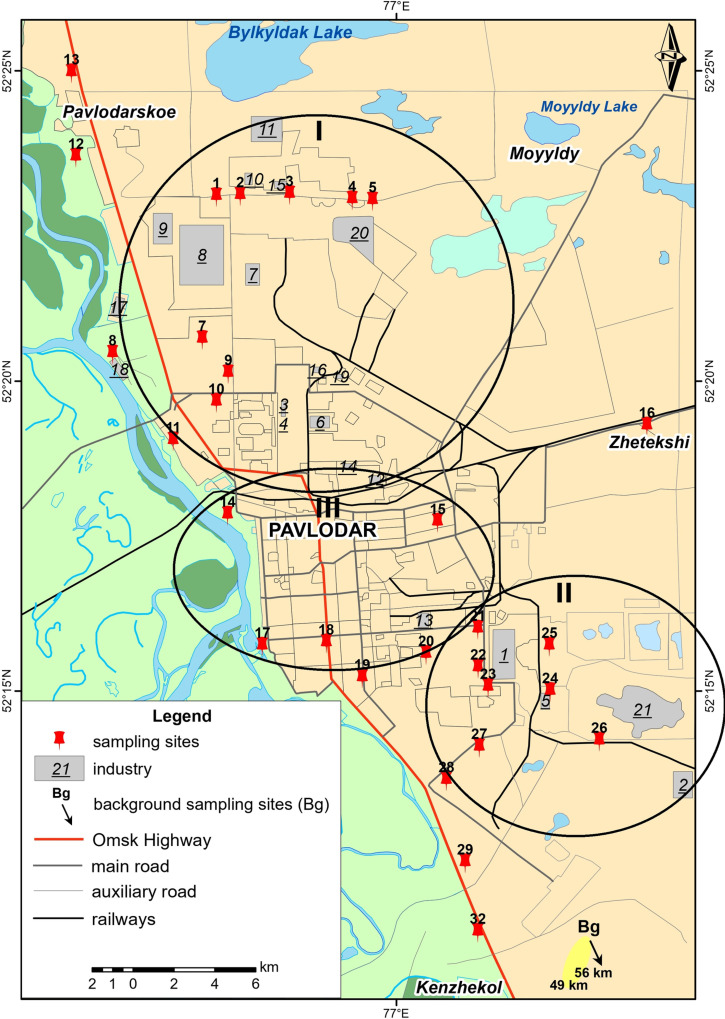
Map of Pavlodar delineating the study area. I – Northern industrial area, II – Eastern industrial area, III – Residential area of the city. 1 - Aluminum of Kazakhstan JSC, 2 - Kazakhstan Electrolysis Plant JSC, 3 - Pavlodar branch of KSP Steel LLP, 4- Pavlodar branch of Casting LLP, 5- CHPP of Aluminum of Kazakhstan JSC, 6- CHPP-2 Pavlodarenergo JSC, 7-CHPP-3 Pavlodarenergo JSC, 8- Pavlodar Petrochemical Plant JSC, 9- Neftekhim Company LTD LLP, 10- Kaustik JSC, 11- UPNK-PV LLP, 12- JSC “Kazenergokabel”, 13- Pavlodar Machine-Building Plant JSC, 14- Pavlodar Pipe Rolling Plant LLP, 15- PMZ “DAMAK” LLP, 16- Cardboard and Ruberoid Plant,17- Pavlodar CGOS, 18- JSC “Pavlodar River Port”,19- LLP firm “Company of Industrial Materials (KPM)”, 20–21 Ash dumps of thermal power plant. The background sites (2) are located near the settlement of Novoyamyshevo (about 50 km from Pavlodar city).

The study of snow cover was conducted in the territory of the city of Pavlodar, which includes five districts: Central, Northern, Eastern, Southern and Western (Fig 2). The northern and eastern districts contain industrial areas. *The northern industrial zone* is home to various industrial enterprises, including KSP Steel LLP, which produces steel pipes for the oil and gas industry, as well as for oil and gas production, geological exploration companies, and machine-building and industrial enterprises in Kazakhstan.

Pavlodar Petrochemical Plant is one of three oil refineries in Kazakhstan and the largest in the northeast of the country, specializing in oil refining and the production of petroleum products. The refinery operates several production units, including primary and deep oil refining, heavy oil residue processing, sulfur production, and general plant facilities. It is also involved in the production of light petroleum products.

The power industry is represented by JSC Pavlodarenergo, which includes Pavlodar CHPP-2 (Combined Heat and Power Plant), Pavlodar CHPP-3, and other facilities. The total installed electric capacity of these power plants is 665 MW, and the installed thermal energy capacity is 1,486 Gcal/hour. In 2021, the total electricity generation by the company was 3,536 million kWh. The primary fuel used at these plants is hard coal from the Ekibastuz Mining site.

Kasting LLP, a steel foundry located in the northern industrial area, produces steel billets, bars, grinding balls, as well as aluminum and lead alloys from ferrous and non-ferrous metals.

The chemical industry in this zone includes JSC “Kaustik,” which manufactures chlor-alkali products, and LLP “Company Neftekhim LTD,” a producer of methyl tert-butyl ether, polypropylene granules, and soft polypropylene packaging.

*In the Eastern Zone,* companies such as JSC Aluminium of Kazakhstan, JSC Kazakhstan Electrolysis Plant, and JSC Pavlodar Machine Building Plant are located. JSC Aluminium of Kazakhstan is the only company in the country producing alumina, a raw material used in aluminum production, with an annual output of 1.4 million tonnes of Al_2_O_3_ alumina. The company sources bauxite and limestone from the Kostanai and Pavlodar regions, and the alumina produced is sent to the Kazakhstan Electrolysis Plant for aluminum metal production. Energy for these operations is provided by Pavlodar CHPP-1. Pavlodar CHPP-1, owned by JSC Aluminium of Kazakhstan and part of the Eurasian Group, was commissioned in 1964. It has an installed electrical capacity of 350 MW and a thermal capacity of 1,182 Gcal [41].

### Sample selection

The selection criteria for the samples were based on the presence of industrial facilities, their type and distance from pollution sources (Fig 2).

Samples were taken at different distances from the industrial facilities and in different directions, using the wind rose (frequency of wind directions) to describe the extent of the facility’s impact on the atmosphere. A total of 32 samples were collected: 14 snow samples in the northern industrial zone, 12 samples in the eastern industrial zone and 6 samples in the residential area of the city.

**Fig 3:**
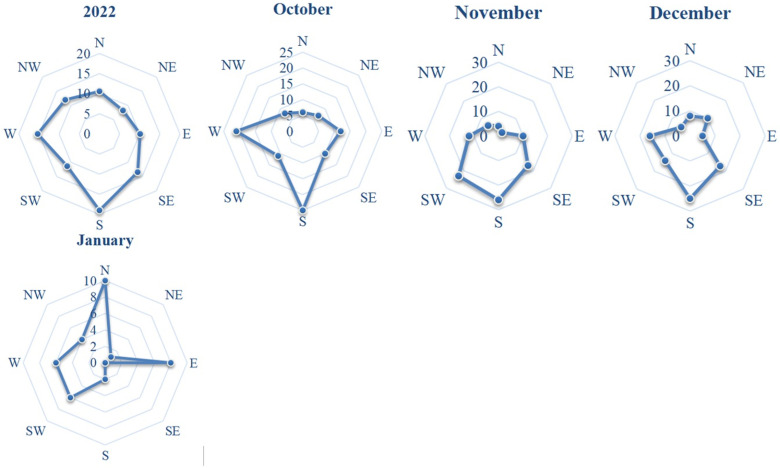
Wind rose for Pavlodar (2022).

The wind rose was generated using data from Kazhydromet for the year 2022 (Fig 3) [33]. The predominant wind direction throughout 2022 was from the south, with significant occurrences from the west and southeast. In addition, winter wind patterns were taken into account during snow accumulation. Stable snow cover was reached on 9 November 2022.

Snow accumulation typically begins in October, when the prevailing winds are from the south and west; in November and December, the prevailing winds are from the south, south-west and south-east.

The environmental gradient was taken into account in the northern industrial zone, reflecting a gradual reduction in the influence of factories, thermal power plants and other facilities. Sampling sites were located at 100 m (site 1), 700 m (site 2), 2000 m (site 3), 2000 m (site 4), 3000 m (site 4), 4600 m (site 5) from the Pavlodar Petrochemical Plant JSC as this facility is significant environmental contributor. In addition to the petrochemical plant, these sites were affected by the activities of the KSP Steel LLP, CHPP-2 and CHPP-3. Sampling locations also included the vicinity of the thermal power plant ash dumps (sites 4, 5).

In the northern industrial zone, the prevailing air masses and associated pollutants were directed away from the city, but extended northwestwards (specifically towards the village of Pavlodarskoye, located 3–5 km from the industrial plant), where samples were collected for analysis (Site 12, 13). Further east of the city, at a considerable distance of 13 km, sampling was carried out in the village of Zhetekshi (site 16). In addition to industrial areas, the samples also included urban gardens sites located in the south direction from the plant at various distances (sites 6, 7, 8, 9, 10).

In the eastern industrial zone, the sampling procedures took into account the wind directions, pollutant transport and deposition within the influence zone of the aluminium smelter and CHPP-1. Samples were collected in all directions from the aluminium smelter and CHPP-1. Samples were taken in south (site 23), east (site 25), southeast (site 24), southwest (site 27), west (site 22) and northwest (site 21) directions from the aluminium smelter.

**Fig 4:**
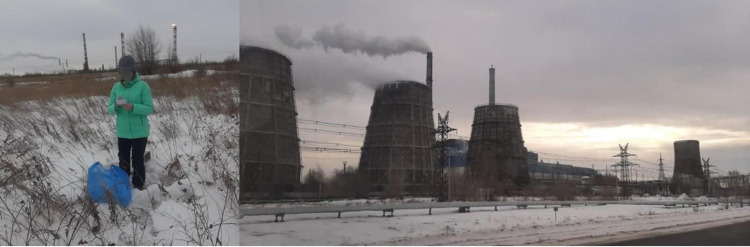
Sample selection. Pavlodar Petrochemical Plant JSC (A); CHPP-3 (B).

Sampling sites also included areas near the thermal power plant ash dumps and sludge fields (site 26), and the village of Kenzhekol (site 32). In addition to industrial areas, the samples also included urban gardens sites located in the south direction from the aluminium smelter (sites 28, 29) (Fig 4).

In the residential area of the city samples were collected throughout the city along main roads (site 18), along the banks of the Irtysh River (sites 14, 17), in areas with private houses, often heated by stoves (sites 15, 19, 2). To reduce the influence of vehicle emissions on the samples, they were collected at a minimum distance of 25 m from the road, as recommended by literature sources [43–45].

Sampling took place in January, when the average snow height was 60 cm. According to observations at the Pavlodar weather station, 9 November is the date of establishment of stable snow cover for 2022, so the snow samples contain three months of precipitation. The number of days from the establishment of stable snow cover is used to calculate the dust load, and the background indicators for the same period are used to calculate the concentration factor.

The background sites, located about 50 km from the city and in the opposite direction to the main wind direction, were minimally affected by industrial emissions. However, despite their distance from the main pollution sources, these sites were not completely free of anthropogenic influences. The proximity of a settlement only 1 km away and the presence of a motorway 500 m from these sites introduced some pollution. This impact is reflected in increased concentrations of pollutants such as suspended particulates and heavy metals from traffic emissions and other sources associated with human activities such as stove heating. Thus, although the baseline sites have been removed from direct industrial emissions, their ecological status is still affected by nearby anthropogenic activities. Nevertheless, these sites remain free of urban industrial pollution and can be used as background points to assess the impact of industry on snow cover pollution.

The sampling method used was the pit method: a depression (depth, width, height) was measured in the snow cover to its full thickness. The snow sampling method (pit method) involved digging pits until the underlying surface was reached, with a depth of 50–60 cm and a width and length of about 20 cm, depending on the thickness of the snow cover. Snow shovels or plastic shovels made of chemically inert materials were used, and surface contaminants were removed before sampling. Each sample weighing at least 6 kg was placed in sealed polyethylene containers and transported frozen.

All snow samples were collected from publicly accessible municipal areas, where no special permits were required for environmental sampling. No samples were taken from private properties, industrial sites, or restricted areas.

### Laboratory analysis

The samples were left in closed plastic containers in the laboratory for 8–12 hours at room temperature (20–22°C) until they had completely thawed naturally. A sample was then taken with a tube from the centre of the meltwater container for further analysis of the liquid phase of the snow. The remaining volume of meltwater was filtered. The meltwater was passed through ashless “Blue Ribbon” filters, which specialize in separating fine-crystalline sediments such as cold-precipitated barium sulphate, copper oxide, etc. from the solution. The pH (acidity indicator) of the unfiltered water was determined using a Checker 1 HANNA-HI 98103 pH meter. Filtered water (1000 ml) was acidified with 5 ml nitric acid. The resulting sediment, after filtration of the meltwater, was dried, weighed and packaged for further analysis to determine heavy metal content [44]. Samples selected for chemical analysis were packaged and prepared for transport, taking into account storage in containers made of chemically neutral material. A total of 64 samples were collected - 32 samples of the solid snow fraction and 32 samples of the liquid fraction.

Laboratory tests were conducted at the Institute of Institute of Radiation Safety and Ecology’s branch laboratory under the RSE at the National Nuclear Center of the Republic of Kazakhstan, Ministry of Energy of the Republic of Kazakhstan. The analysis of the chemical element content in the solid snow phase was carried out by inductively coupled plasma mass spectrometry using an Agilent 7700 X ICP-MS, in accordance with measurement procedure No. 499-AES/MS The Methodology of Quantitative Chemical Analysis “Methods of quantitative chemical analysis. Determination of the elemental composition of rocks, soils, and bottom sediments by atomic emission with inductively coupled plasma and mass spectral with inductively coupled plasma methods” KZ.07.00.03351–2016. Thirteen elements were selected for further processing, including the most toxic and significant contributors to snow pollution in the city: V, Cr, Mn, Co, Ni, Cu, Zn, As, Sr, Mo, Cd, Ba, Pb, with detection limits ranging from 0.005 to 0.1 µg/l and uncertainties of 10–15%.

The samples of solid snow sediment were air-dried until they reached air-dry weight. The air-dry samples were sieved through a polyamide sieve with an aperture diameter of 1 mm. A sample weighing 200 g was selected using the quartering method and ground in a disk mill “Pulverisette 9” (hardened steel set) for 20 minutes at a rotation speed of 1000 rpm. A further 50 g aliquot was selected from the ground homogeneous sample using the quartering method and additionally ground for 20 minutes.

Acid decomposition of the samples was carried out according to methodology No. 499-AEC/MS MKHA GSI RK under No. KZ.07.00.03351–2016 in Teflon cups. The aliquot of each sample is 0.1 g. Together with the analyzed samples, one control sample, one standard sample, and one blank sample were decomposed. In each cup, before the start of the decomposition, a solution of Nd, Dy, Yb with concentrations of 80, 50, and 30 μg/L was added and the cup was placed on a plate at a temperature of 130 °C. Then, concentrated acids HF, a mixture of HF:HNO₃ (3:1), HCl, and HNO₃ were sequentially added to the cups and evaporated to wet salts. Afterwards, all samples were brought to 3 M HNO₃, closed with Teflon lids of the “hourglass” type, and heated for 30 minutes. Then the lids were removed and the solutions were evaporated until intense white vapors appeared upon heating to 170–180 °C. The cups were cooled, and their walls were washed with deionized H₂O. The resulting solutions were again evaporated to wet salts. Then, HCl and H₃BO₃ were added to each cup and the solutions were evaporated to a volume of approximately 0.7 ml. The obtained solutions were transferred to flasks with the addition of an internal standard In (1 μg/L) and brought to the required volume with deionized H₂O. Before carrying out the measurements, all solutions were diluted 10-fold with deionized H₂O for laboratory analysis.

The analysis of the chemical element content in the liquid phase of snow was carried out according to the GOST standard ISO 17294-2-2019 Water Quality Standards [46]. Nine elements were selected for processing from meltwater or the liquid phase of snow: V, Cr, Mn, Cu, Zn, As (metalloid), Sr, Ba, Pb with detection limits ranging from 0.005 to 0.1 µg/l and uncertainties of 10–15%.

#### Equipment calibration and quality control measures.

10 µg/L and 20 µg/L calibration solutions of the analytes were used to calibrate the mass spectrometer. Multi-element standard solutions of standard samples (SS) of metal composition produced by Perkin Elmer (USA) were used for calibration curves: № 9300231, № 9300233, № 9300235 with nominal certified value of metal content 10 mg/l with uncertainty of certified value 0.5% (dilution factor k = 2).

Quality control of the measurements was performed by measuring the calibration solution in every 10 samples. Inorganic Ventures IV-ICP-MS-71A, CMS-1 (Inorganic Ventures, USA) is used for the preparation of samples to check the correctness (confirmation) of the calibration characteristics. In case of an unsatisfactory calibration result (8–10% deviation of the calibration curve), the instrument was recalibrated using new background parameters.

The quality control of the measurements in the analysis of solid samples was carried out as follows: blank samples (BLK) were prepared by following all the steps of sample preparation, but without adding the sample. At the same time, laboratory control samples for analytical process control (LCS) were prepared using all steps of sample preparation according to the respective analytical procedure.

### Statistical processing and coefficient calculations

Statistical analysis of the data was performed, and concentration coefficients for various indicators were calculated, including pollutant concentration factor (Kc), pollution index (Zc), dust load (Pl), total load (Ptotal), element contamination load (Kl), total load indicator (Ztotal) [44], and phase distribution coefficient (Kpd) (partition coefficient) [47].

The concentration coefficient (Kc) of a chemical element was calculated by comparing its actual (anomalous) content (C) to its background concentration (Cbg):


$$\begin{array}{c} Kc=\frac{C}{Cbg}, \end{array}$$ (1)

The pollution index (Zc) equals the sum of concentration coefficients of chemical elements whose content exceeds background values and is expressed by the formula:


$$\begin{array}{c} Zc=\sum Kc-\left(n-1\right), \end{array}$$ (2)

where n - is the number of anomalous elements considered.

The phase distribution coefficient (Kpd), representing the ratio of insoluble to soluble forms of element compounds:


$$\begin{array}{c} Kpd=\frac{Cs}{Cl}, \end{array}$$ (3)

where C_s_ - is the concentration of the substance in the solid phase (snow filtrate), and C_l_ - is the concentration of a substance in the liquid phase (meltwater). The scale for interpreting the obtained values of the phase distribution coefficient is given in S1 Table.

The dust load (Pl) in a snow sample, measured in mg/ m²/day, represents the amount of solid fallout per unit area over a specific time period. It is calculated as:


$$\begin{array}{c} Pl=\frac{P}{S\text{*}t}, \end{array}$$ (4)

where P - is the mass of dust in the sample (mg), S - is the pit area (m²), and t - is the time from the beginning of snowfall (number of days).

The element contamination load on the environment, representing the mass of pollutant falling per unit area per unit time, was also computed, considering both the dust load (Pl) and the element concentration in the snow (C). This led to the calculation of:

1) the total load generated by the release of a chemical element into the environment:

$$\begin{array}{c} Ptotal=C*\frac{\text{P}}{100}, \end{array}$$ (5)

2) element contamination load (Kl), the coefficient of relative increase in the total element load:

$$\begin{array}{c} Kl=\frac{Ptotal}{Pbg}, \end{array}$$ (6)

$$\begin{array}{c} Pbg=Cbg*Plbg, \end{array}$$ (7)

where Cbg - is the background content of the element under study, Plbg is the background dust load, and Pbg - is the background load of the element under study.

Since man-made anomalies often have a multi-element composition, total load indicators (Ztotal) were computed to characterize the combined effect of exposure to a group of elements:


$$\begin{array}{c} Ztotal=\underset{i=1}{\overset{n}{\sum }}\;Kl-\left(n-1\right), \end{array}$$ (8)

where n - is the number of elements considered [39].

#### Potential ecological risk (RI).

The Potential Environmental Risk (RI) is an index used to assess the degree of environmental risk caused by heavy metal concentrations in soil. This index was introduced by Håkanson, 1980 and is calculated using the following formula


$$\begin{array}{c} \begin{array}{c} RI=\underset{i=1}{\overset{n}{\sum }}\;E_{r}^{i} \end{array} \end{array}$$ (9)

where n is the amount of heavy metals and $E_{r}^{i}$ is the unit index of the environmental risk factor calculated by the equation


$$\begin{array}{c} \begin{array}{c} E_{r}^{i}=T_{r}^{i}*PI \end{array} \end{array}$$ (10)

where $T_{r}^{i}$is the toxicity response coefficient of a single metal and PI is the calculated value of the single pollution index [48].

Analysis of Variance (ANOVA) was conducted to examine the mean significance difference in the studied heavy metal concentrations across different regions (northern industrial, eastern industrial and residential zones) and also between snow and melted snow water. F-values were determined for the comparison of variance between and within different groups, while p-values were utilized to determine the significance level.

### Cartographic representation

The cartographic material presented here has been produced by interpolation using the ArcToolbox tools of the ArcGIS 10.1 software. For this study, the Spline tool was selected, which interpolates the raster surface based on point values using a 2D curvature-minimising spline method. The resulting smooth surface passes directly through the input points [49].

## Results and discussion

### Content of heavy metals in solid snow sediment in Pavlodar City

When studying heavy metal contamination in the snow cover of the city of Pavlodar, we determined the concentrations of trace elements and compared them with values reported in other urban areas, as well as with previous studies conducted on the snow cover of Pavlodar. Statistical analysis, background concentration and concentration coefficient (Kc) of the trace elements in the snow filtrate in Pavlodar is given in S2 Table.

Overall, for Pavlodar city, the descending order of mean metal concentrations in snow is as follows:

Ba (949.4)> Mn (638.1)> Cr (346.9)> Zn (274.6)> Sr (263.7)> Cu (121) > V (114)> Pb (114)> Ni (28.3)> As (12.6)> Co (10.1)> Cd (4.1)> Mo (2.2). Comparison with other studies showed that the concentrations of metals (copper, nickel, cadmium, cobalt, manganese and zinc) in Severodvinsk are on average higher than in Pavlodar [24] (Yakovlev, 2022). In particular, the level of manganese in Severodvinsk is 1071 mg/kg, while in Pavlodar it is 638.1 mg/kg, and zinc is 423.5 and 274.6 mg/kg, respectively. In studies around an electrometallurgical plant in the Kaluga region, zinc and copper concentrations were found to be higher than our data [50]. Research carried out in the city of Pavlodar in 2001–2002 also focused on the study of heavy metals in the snow cover [31]. According to the results of this study, the average content of microelements in snow is as follows Sr (266)> Zn (264.3)> Mn (171.2)> Cu (137.4)> Pb (102.5)> Ni (77.9)> Cr (56.2) > V (55)> Co (41.6)> Mo (2.2)> Cd (2.1). If we compare these data with our own, we find that the situation has deteriorated for most elements over more than 20 years (Table 1). The content of chemical elements in liquid and solid fractions was determined by the atomic absorption method on a Perkin Elmer model 403 spectrophotometer with an HGA-74 electrothermal analyser and a deuterium background corrector in previous study [31]. The fact that we have used the more sensitive method of inductively coupled plasma mass spectrometry in our study makes it possible to make an approximate comparison of the data, which may be useful in identifying general trends and tendencies. However, it is important to take into account possible uncertainties and differences in accuracy between methods.

**Table 1:** Comparison of heavy metals content in snow of our research and literature data [31].

Microelements	Heavy metals content in snow, mg/kg	
	Our data	[31]
Mn	638.1 ± 48.9*	171.2 ± 20.7
Cr	346.9 ± 48.4	56.2 ± 3.1
Zn	274.6 ± 45	264.3 ± 24.1
Sr	263.7 ± 12.2	266 ± 23.7
V	114 ± 19.9	55 ± 3.4
Pb	114 ± 15	102.5 ± 8.0
Cu	121 ± 17.8	137.4 ± 13.9
Ni	28.3 ± 1.7	77.9 ± 5.3
Co	10.1 ± 0.6	41.6 ± 2.9
Cd	4.1 ± 1.3	2.1 ± 0.26
Mo	2.2 ± 0.2	2.2 ± 0.22

* Standard error of the mean.

Chromium has increased the most, with a 6-fold increase compared to our data, followed by manganese - 3.7-fold, cadmium and vanadium - 2-fold. Strontium, copper, lead, zinc and molybdenum remained relatively stable with minor changes, while cobalt concentration decreased by a factor of 4.

Series of coefficients of variation: Cd (175) > V (99) > Zn (93) > Cu (83) > Cr (79) > Pb (75) > As (48) > Mn (43)> Ba (36) > Co (36) > Mo (36) > Ni (34) > Sr (26).

The concentration coefficient, which indicates the excess of the element content compared to the background, according to this study, follows this series: Cd(5.2) > Pb(2.7) > Mo(2) > As(1.8) > Cu(1.7) > V(1.7) > Zn(1.6) > Co(1.4) > Ni(1.4)> Sr(1.3) > Mn(1.13) > Ba(0.95) > Cr(0.5). The concentration coefficient ranged from 0.5 to 5.2, with an average of 1.8. The metal content in the city is not significantly higher than that outside the city. This could be due to the fact that the background areas were outside the city but influenced by anthropogenic activities, such as the proximity of a populated area (1 km) and the location of the motorway 500 metres away. This helps us to determine the impact of industrial activity on snow cover.

Since the background values of our study were high due to the proximity of the settlement, we used for comparison the background values of heavy metal concentration in snow obtained in previous studies [31] conducted in the territory of the city of Pavlodar.

The background sites of the study were located 80 km from the city in the opposite direction of the wind rose, where there were no anthropogenic sources of pollution. By comparing our concentrations with the background data, the following results were obtained (Table 2).

**Table 2:** Comparison of concentration coefficients of heavy metals in snow.

Microelements	Concentration coefficients (Kc)	
	Our data	[31]
Mn	1.13	26,3
Cd	5.2	26,0
Cr	0.5	18,9
V	1.7	11,6
Sr	1.3	8,9
Mo	2	7,6
Pb	2.7	6,1
Cu	1.7	5,9
Zn	1.6	5,7
Ni	1.4	1,3
Co	1.4	1,3

The series of concentration coefficients based on literature sources of background concentrations has the following form Mn(26,3) > Cd(26) > Cr(18,9) > V(11,6) > Sr(8,9) > Mo(7,6) > Pb(6,7) > Cu(5,9) > Zn(5,7) > Ni(1,3) > Co(1,3).

The levels of magnesium and cadmium in urban snow are 26 times higher than the background level. This is followed by chromium and vanadium, with levels more than 10 times higher, and the lowest levels of nickel and cobalt, with urban levels only 1.3 times higher than the background level.

So the picture is quite different. If we compare how many times the concentration at the background site with anthropogenic influence (40 km) differs from the concentration at the background site without anthropogenic influence (80 km), we obtain the following data. The difference in the concentration coefficient is 37.8 times for chromium and 23.3 times for manganese, i.e., these metals are a direct indicator of pollution from anthropogenic sources, industry and traffic. Vanadium and strontium (7 times, cadmium 5 times) follow in descending order. The concentration coefficients of nickel and cobalt are practically the same in both studies, indicating background pollution by these metals.

### Spatial distribution of heavy metals in solid snow sediment

A comparative physical and chemical characterisation of heavy metals in solid snow sediments was carried out in different urban districts to determine the spatial distribution of contamination (Table 3). The toxicity class of the heavy metals was taken into account when analysing the data. Toxicity class 1 includes Zn, As, Cd, Pb; toxicity class 2 includes Cr, Co, Ni, Cu, Mo; and class 3 includes V, Mn, Sr, Ba [51]. Comparative characteristics of heavy metals in solid snow sediment by city districts is given in S3 Table.

**Table 3:** Analysis of variance illustrated the mean significance difference among the northern industrial, eastern industrial and residential zones Pavlodar, Kazakhstan.

Variables	Sum Sq	Mean Sq	F-value	p-value
As	158.35	79.173	2.2986	0.1184
Ba	626100	313050	2.9729	0.06692
Cd	344.48	172.238	3.8682	0.03243
Co	86.63	43.315	3.7951	0.03435
Cr	56663	28332	0.3629	0.6988
Cu	6010	3004.8	0.2813	0.7569
Mn	6589	3294	0.0404	0.9605
Mo	3.4251	1.71256	3.0905	0.06071
Ni	673.43	336.72	4.3149	0.02289
Pb	71942	35971	1.1519	0.3301
Sr	3267	1633.3	0.3292	0.7222
V	24659	12329	0.9643	0.3931
Zn	290844	145422	2.444	0.1045

The average content of trace elements in the northern industrial zone shows high levels of zinc (286.2 mg/kg) and lead (158.5 mg/kg). In the eastern industrial zone, zinc (187.5 mg/kg) and cadmium (8.84 mg/kg) are also prominent among the elements of the first toxicity class. Concentrations of Zn, Cr, and Pb in the solid phase of snow in residential areas of the city exceed those in industrial zones, amounting to 436.6, 259.1, and 218.6 mg/kg, respectively.

The coefficient of variation shows the degree of variation in the concentrations of elements in the snow. There is a significant variability in the content of elements in the northern industrial zone; the coefficient of variation is greater than 50% in all cases. The largest variation in element content in the eastern industrial zone is observed for Cd - the coefficient of variation is 118%. In the residential areas, the largest variation corresponds to lead, zinc and copper, while the smallest variation corresponds to vanadium.

The coefficient of concentration was then calculated in relation to the background level of the substance. The background indicators had an anthropogenic influence, but without the direct influence of urban industry. As a result, the concentration coefficients in the northern industrial zone are low, ranging from 0.4 to 3.7. In the eastern industrial zone the coefficients are slightly higher, ranging from 0.3 to 11.1. Thus, the analysis of the data shows that the pollution of the environment with different heavy metals in the city is associated with both industrial processes and transport activities, and that the level and nature of the pollution varies according to the specific zone. Thus, to assess the impact of the Pavlodar Petrochemical Plant and other industrial facilities (the northern industrial zone) on the content of heavy metals in snow, significant exceedances of background concentrations of manganese, chromium, vanadium and other metals were detected. The distribution of metals in the snow cover showed that the elements were high near the oil refinery and decreased at a distance of 700 metres. The possible influence of other industries and the wind direction from CHPP-3 are also noted. The levels of elements decrease with distance from the pollution sources. However, manganese and barium show more stable values, while chromium, zinc, lead and cadmium show increased contents in the vicinity of Kaustik JSC (chemical production). The total content of manganese, strontium, cobalt and arsenic varies insignificantly at all sites, indicating uniform pollution by these substances. Lead levels are highest in industrial areas and in settlements near motorways.

The highest levels of heavy metals in snow from the eastern industrial zone of the city are barium, manganese, chromium, strontium and zinc. Studies have shown that the content of heavy metals in snow increases in the north-eastern direction from industrial enterprises in the eastern industrial zone of Pavlodar. Pavlodar, which may indicate the deposition of pollutant particles due to prevailing winds or cross-contamination from other industrial facilities.

The concentration of heavy metals in the snow of the residential zone of the city is slightly lower than in all other zones. The highest concentration factor in residential areas is lead - 5.1 times higher than the background level and zinc - 2.5 times higher.

Based on analysis of variance (ANOVA), significant differences were observed in cadmium (p = 0.032), cobalt (p = 0.034) and nickel (0.022) heavy metal concentrations among the northern industrial zone, eastern industrial zone and residential regions (Table 3). It indicates that the concentration of these heavy metals is significantly influenced by the tested factors. The barium and molybdenum metals comprehended mean marginal significance level with p-value 0.06. Whereas, As, Cr, Cu, Mn, Pb, Sr, V, and Zn potential toxic elements do not show mean significant difference based on ANOVA.

### Dust deposition on snow cover

**Fig 5:**
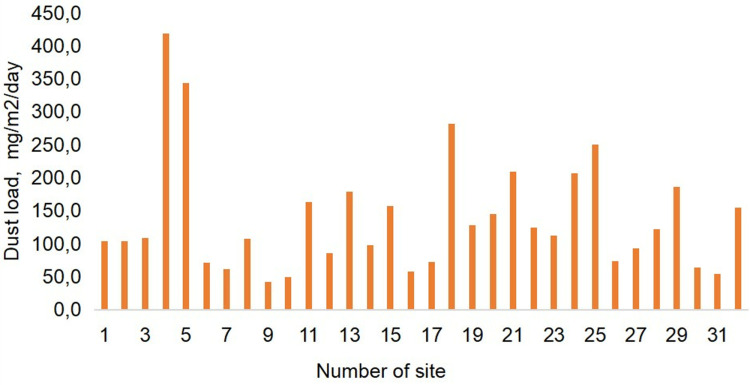
Dust load on snow cover in Pavlodar. The mean value of dust load in the city is 138.4 mg/m2/day, SD 87.3 (standard deviation).

Dust load serves as a control parameter, representing the mass of dust deposited in a given area per day or per year. Dust load values in Pavlodar ranged from 42.3 to 418.5 mg/m2/day (Fig 5). In residential areas, dust deposition values ranged from 72.6 to 282.2 mg/m2 per day. The lowest values were recorded at a remote sampling site located within the water protection zone of the Irtysh River (site 17), while the highest dust levels were observed at the intersection of major city roads (site 18). Within the eastern industrial zone (comprising the aluminium smelter and the thermal power plant), the dust load varied from 73.8 to 250 mg/m2 per day, with the highest concentration in the eastern part of the aluminium smelter (site 26) and the lowest concentration south of the ash dump (site 25). The reduced dust load near the ash dump can be attributed to the prevailing wind directions, mainly from the south-east and south.

In the northern industrial zone, dust loads ranged from 42.3 mg/m2/day at dacha (garden) sites (site 9) to 418.5 mg/m2/day at the northern part of the thermal power plant ash dumps (site 4). Sites 30 and 31 represent background levels, with dust loads of 63.7 and 53.6 mg/m2/day, respectively.

The total pollution load of the trace elements studied corresponds to the distribution of metals by content (Table 4). If we consider the pollution load (the content of elements in dust), the distribution is generally as follows Cd(13.9)>Pb(5.3)>Mo(4.8)>As(4.4)>Zn(4.1)>Mn(3,7)>V(3.9)>Cu(3.7)>Mn(3.7)>Ni(3.4)>Sr(3)>Ba(2.2)>Co(1.9)>Cr(0.9).

**Table 4:** Characteristics of pollutant loads in dust deposition in Pavlodar.

Trace element	Concentration, C, mg/kg	Element concentration in dust. Total pollution load, (Ptotal), mg/m2/day (dust deposition)	Background pollution load, (Plbg), mg/m2/day (dust deposition in the background area)	Element contamination load (Kl)
Zn	274.6 ± 45*	408.5	100.5	4.1
As	12.6 ± 1.1	18.2	4.1	4.4
Cd	4.1 ± 1.3	6.3	0.45	13.9
Pb	114 ± 15	129.6	24.5	5.3
Cr	346.9 ± 48.4	407.1	439	0.9
Co	10.1 ± 0.6	14.6	7.5	1.9
Ni	28.3 ± 1.7	40.7	12.0	3.4
Cu	121 ± 17.8	155.6	41.6	3.7
Mo	2.2 ± 0.2	2.9	0.6	4.8
V	114 ± 19.9	148.2	37.6	3.9
Mn	638.1 ± 48.9	881.9	237.5	3.7
Sr	263.7 ± 12.2	367.3	120.6	3
Ba	949.4 ± 60.9	1294.5	586.6	2.2

* Standard Error of the Mean.

By comparing the average heavy metal content in the solid phase of the snow cover with background values, zonal technogenic anomalies can be identified at Pavlodar (Table 5).

**Table 5:** Dust load and total load indicator of the districts of Pavlodar.

Area	Volume of dust deposition (dust load), Pl	Total load indicator (Ztotal)
Background dust deposition	58 ± 7.1	2.5
Northern industrial zone	135.5 ± 112.3	33.9
Eastern industrial zone	152.3 ± 54.8	64.6
Residential area of the city	147.1 ± 73.1	41.1
City average	138.4 ± 87.3	47.9

According to the guidelines [39], the total heavy metal load in Pavlodar is low, possibly due to the height of the snow cover, but the high metal content in the snow may be due to the chemical composition and distribution in the environment [15].

Spatial analysis of dust loads in the city of Pavlodar showed that the highest levels of chromium, zinc and lead indicate that motor traffic is the main source of pollution, while the eastern industrial zone has high levels of manganese, and the northern industrial zone has high levels of cadmium and barium, probably related to the characteristics of industrial production (Table 5). Urban areas often have elevated levels of lead (Pb) due to a variety of factors, including past industrial activity, leaded petrol and lead-based paint. For example, studies have shown that areas near major highways, which are significant sources of vehicle emissions, tend to have higher lead concentrations. This correlation has been documented in various urban settings, including Yogyakarta, Indonesia, where lead levels were found to be significantly elevated in residential areas near busy roads [52]. In addition, the historical use of leaded petrol has left contamination, particularly in urban areas where the practice was common until the mid-1980s [53,54]. The sites with elevated lead concentrations are closely connected to the main road, so the contamination is the result of anthropogenic activities.

The average estimate of Pb in residential soils is three times higher in urbanised areas than in non-urbanised areas. Urbanised areas may have higher soil lead loads because urban centres tend to be older and have denser development and traffic, which can lead to increased lead in soil over time from gasoline and lead-based paint. In addition, urban areas may have contributions from brownfield sites and historic industrial activities that contribute to elevated levels.

One of the main sources of lead pollution in the refining industry is the combustion of fossil fuels in refining processes. The flaring of associated gas and other hydrocarbons releases not only carbon dioxide but also heavy metals, including lead [55]. This pollution can lead to lead deposition in neighbouring areas, affecting both soil and water quality. For example, studies have shown that areas near artisanal refineries have elevated concentrations of lead and other toxic substances, which can have detrimental effects on local flora and fauna [56].

The sources of heavy metals in energy production facilities are multifaceted, mainly industrial processes such as coal combustion, metal processing and the use of various chemicals in energy production [57–59]. For example, coal-fired power plants are notorious for releasing heavy metals such as mercury, arsenic and lead into the atmosphere and surrounding ecosystems, which can subsequently contaminate water sources and soil.

The metallurgical industry is a major contributor to heavy metal pollution, which poses serious environmental and health risks. Heavy metals such as lead, cadmium, arsenic and chromium are commonly released during various metallurgical processes, including mining, smelting and metal finishing [60–62].

According to regulatory data, Pavlodar has a low level of dust pollution [44]. Previous studies of the daily dust load in Pavlodar and its suburbs showed that it is 9.75 kg/km2/day, which corresponds to the norm of aerosol particle fallout for flat continental territories of temperate latitudes [32]. Comparing the dust load indicators with the literature data, it turned out that the pollution in the CHP area exceeds our values by 3–4 times on average [63]. However, as for residential areas, our data are more than 2 times higher than the literature (63 mg/m2/day) in a residential area [64–66].

### Content of heavy metals in meltwater of Pavlodar City

As mentioned above, our study divided snow into two phases: solid - remaining after filtering meltwater, and liquid - filtered meltwater. In the meltwater or liquid phase of the snow, the content of 9 elements was analysed: V, Cr, Mn, Cu, Zn, As (metalloid), Sr, Ba, Pb, which are highly toxic and contribute significantly to the pollution of the city’s snow cover [67]. Metals such as cadmium, cobalt, nickel and molybdenum, which are present in the analysis of the solid phase of snow, were not considered because their content in meltwater is either undetectable or present in very low concentrations. In the city, the concentration of heavy metals shows the following pattern. Metal content in µg/l: V - 1.3–2.7, Cr - 0.4–3.8, Mn - 9–72, Cu - 1.1–8.8, Zn - 1–250, As - 1–18, Sr - 12–160, Ba - 7.3–79, Pb - 2–13. The series of metal concentrations according to the average element content is as follows Zn 58.6 > Sr 34.8 > Mn 26 > Ba 21.9 > V 5.1 > Pb 4.7 > Cu 4 > As 3 > Cr 1.9. Thus, it was found that the zinc and strontium contents predominate in the liquid fraction of the snow in the city. Comparing our data with literature sources (Table 5), it can be observed that our data exceed the urban concentrations of metals in European cities by 4–5 times [68]. Comparisons of chemical concentrations in snow under various conditions are shown in S4 Table.

After comparing the results of many studies on the content of heavy metals in snow, it was found that the level of contamination of the snow cover in the city of Pavlodar corresponds to the level of contamination typical of industrial areas.

To assess the levels of spatial pollution with heavy metals in various zones of the city of Pavlodar, a table was prepared that included decreasing series of the average content of metals in meltwater, as well as coefficients of variation and concentration coefficients (S5 Table).

The average metal concentrations in snow indicate that zinc content (32.6 µg/l) is most prevalent in the residential area of the city. The eastern industrial zone is most polluted with manganese, with a content of 28.3 µg/l. In the city centre, in residential areas, the picture was the same as throughout the city. The chromium concentration coefficient in the city and in all industrial zones exceeds the background level by 4.4 times in the city as a whole; in the eastern industrial zone, the coefficient is 3.5, and in the northern industrial zone - 3.4. Lead exceeds background concentrations in a residential area of the city by 2.8 times.

### Comparison of heavy metals concentration in snow and melted water based on ANOVA

Analysis of variance showed mean significant difference between snow and melted snow water for all the studied heavy metals in the studied region (Table 6). The most significant variation was observed for Mn, Ba, Sr, Cr, and Cu metals. It indicates that heavy metal concentrations in snow and melted water were significantly influenced by environmental factors.

**Table 6:** ANOVA showing the mean significant differences of heavy metals between snow and melted water of snow in the Pavlodar, Kazakhstan.

Variables	Sum Sq	Mean Sq	F value	p-value
V	185301	185301	27.025	2.824E-06***
Cr	1494699	1494699	43.53	1.485E-08***
Mn	5635951	5635951	135.79	<2.2e-16***
Cu	214222	214222	39.274	5.238E-08***
Zn	927203	927203	25.766	4.406E-06***
As	1532.8	1532.77	64.62	5.928E-11***
Sr	790926	790926	263.11	<2.2e-16***
Ba	12628165	12628165	196.72	<2.2e-16***
Pb	304028	304028	18.114	0.00007846***

### The ratio of metal content in the soluble and insoluble fractions of snow and the acidity of melt water

The phase distribution coefficient was calculated for the concentrations of metals in Pavlodar in different phases. For all elements studied, a high (Mn, Pb, V, Cu) or very high (Cr) predominance of suspended insoluble forms was revealed, with the subsequent deposition of these particles in the soil. Moreover, the maximum number of solid particles was noted for chromium, which exceeded dissolved particles by 280 times. It was observed that the concentrations of suspended and dissolved forms of zinc (1.4) and strontium (1.4) were approximately equal, while the concentration of arsenic was the lowest.

The pH value of the snow cover in the city is within the range of 5.3–7.1. The eastern industrial zone, which is associated with the activities of the aluminium smelter, exhibits a slight excess. The atmosphere in residential areas is less polluted, and the pH of snow water is closer to the pH of pure atmospheric precipitation, with values ranging from 5.5 to 6.4. The maximum pH values, 7 and 7.1, are observed in the zone affected by emissions from aluminium production and CHPP-1.

### Cartographic representation of heavy metal pollution distribution in Pavlodar City

**Fig 6:**
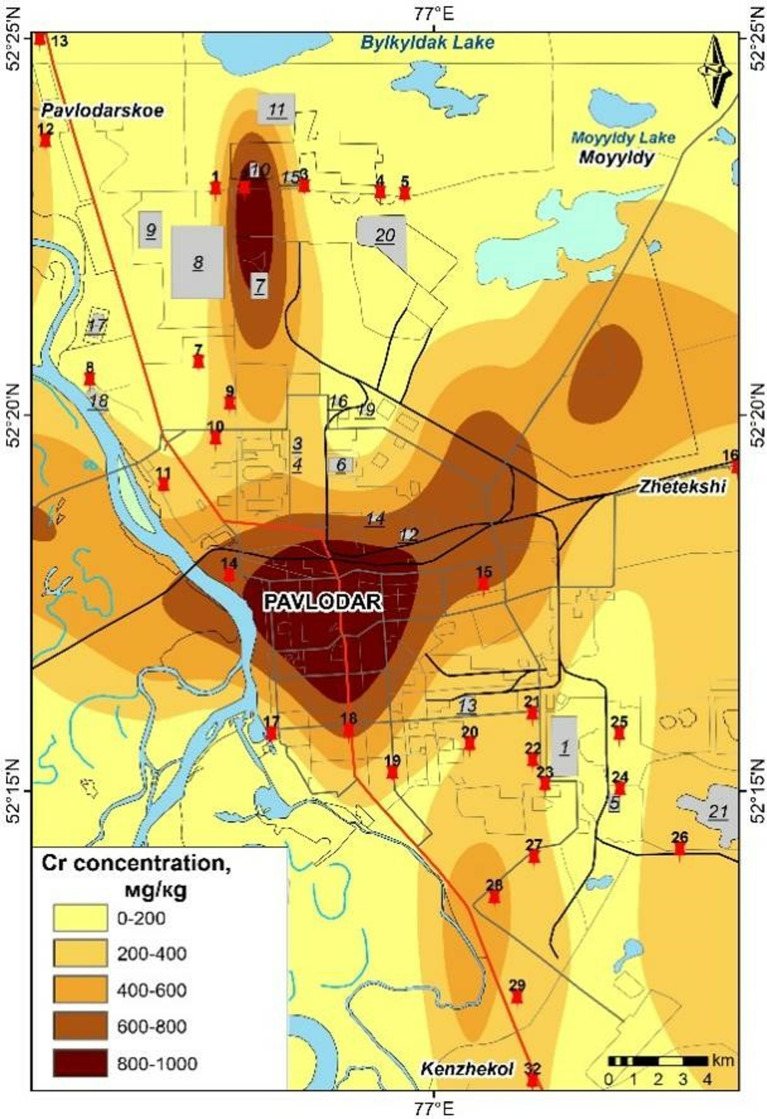
Content of Cr in the solid phase of snow in Pavlodar [69].

**Fig 7:**
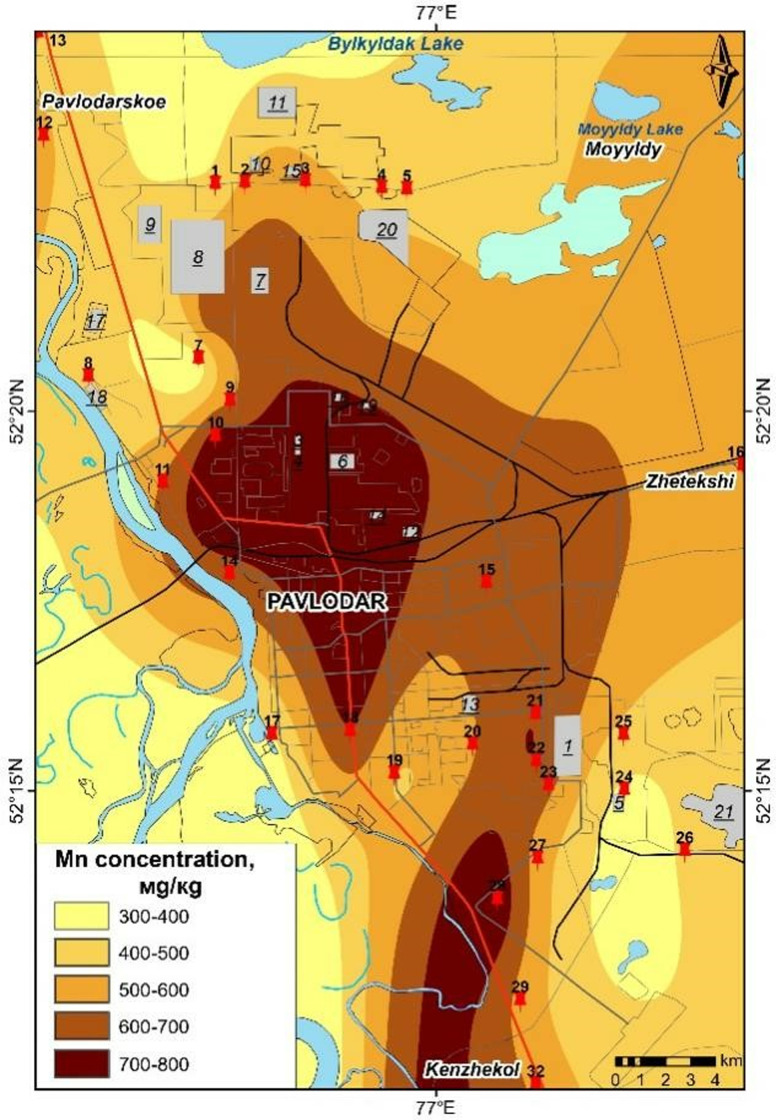
Content of Mn in the solid phase of snow in Pavlodar [69].

**Fig 8:**
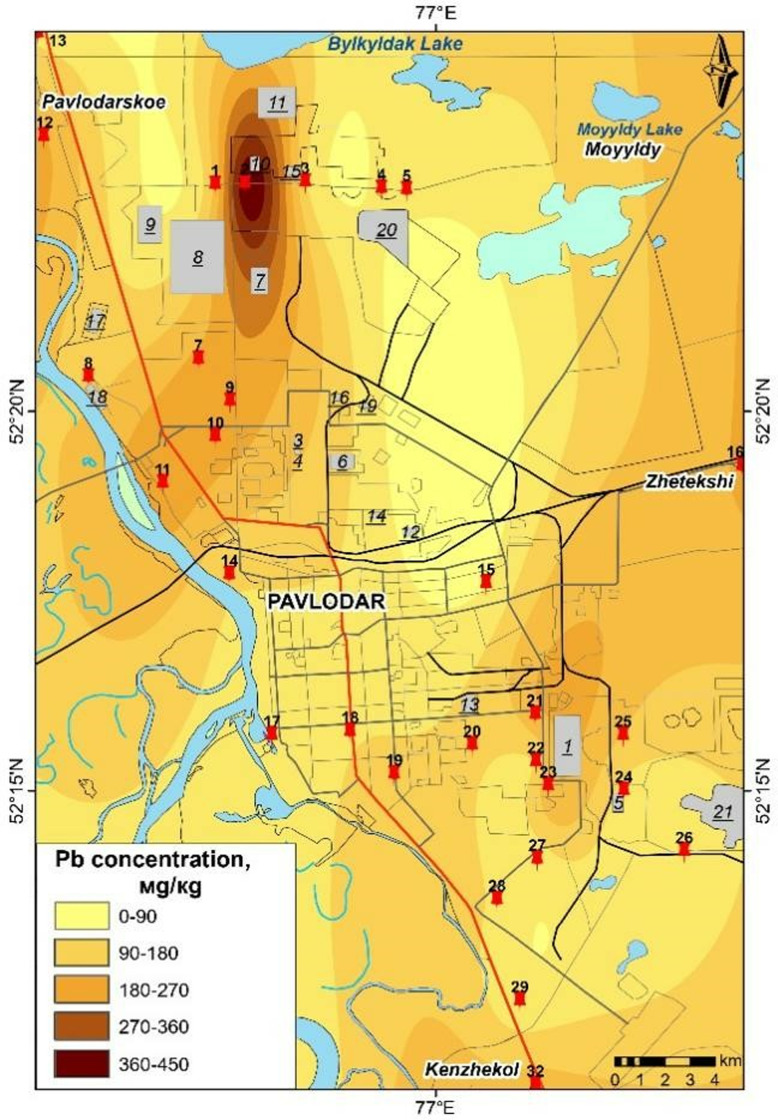
Content of Pb in the solid phase of snow in Pavlodar [69].

**Fig 9:**
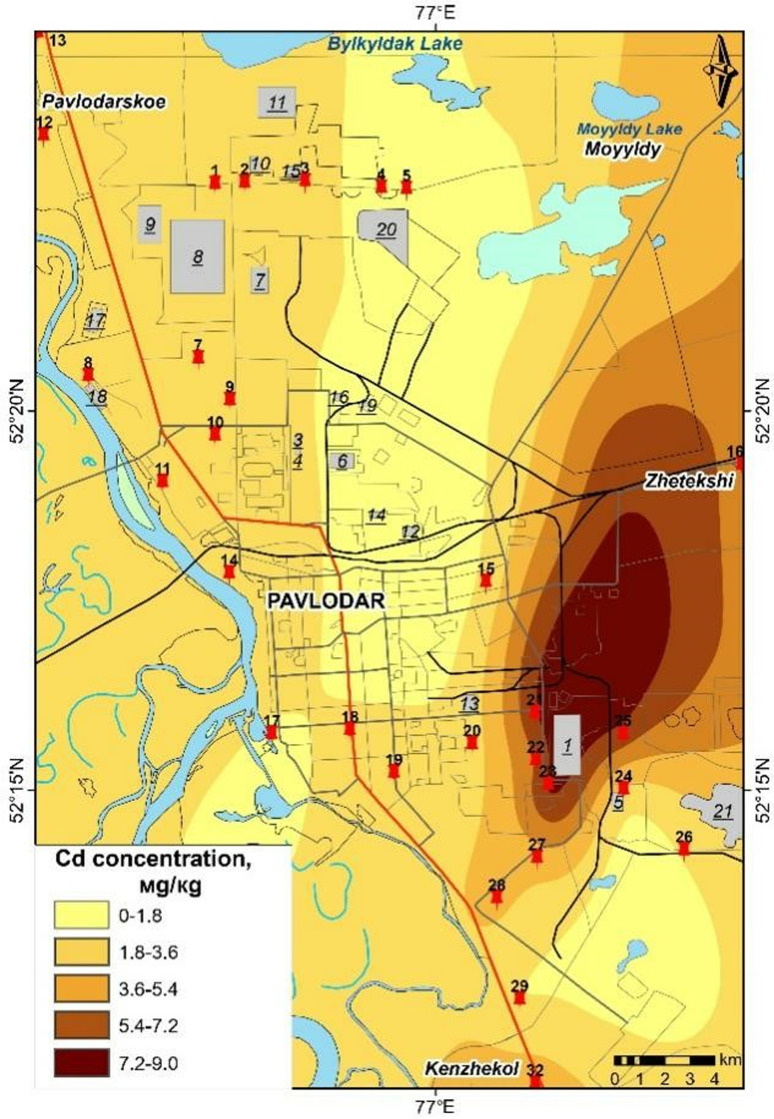
Content of Cd in the solid phase of snow in Pavlodar [69].

The maps presented in Figs 6–9 were generated based on the average concentrations of heavy metals in solid snow sediment. The data was extrapolated using a process of interpolation. The cartographic analysis revealed that among the most toxic metals, cadmium, chromium, and manganese are most closely linked to industrial facilities, roads, etc.

Cadmium levels remain persistently elevated across the city, including in industrial zones, with particularly elevated concentrations observed in the eastern industrial zone. The northern industrial zone and urban areas exhibit elevated levels of chromium, while lead is present in proximity to petrochemical plants and thermal power plants. Chromium pollution is present in the vicinity of the plants along transport routes, even in garden plots. Manganese pollution is concentrated in the city centre, with a gradual decrease in concentration to the north.

It is notable that the peak concentrations of trace elements in snow are the result of a complex interplay of factors, including meteorological conditions and the emission of these elements from a multitude of sources. A spatial analysis of emission pollution reveals the accumulation of local pollutants in proximity to emission sources [68,70] identified industrial hubs of pollution in Pavlodar city through research. The maximum element content in soil is not associated with a single centre, but is concentrated in several hubs with varying element compositions and accumulation levels.

### A sanitary and hygienic evaluation of snow cover contamination

In order to assess the potential impact of heavy metals on human health, we conducted an examination of the maximum permissible concentration of elements (MPC) in soil, as there are no separate standards for snow cover. As our study focused on solid snow sediment following filtration of melt water, we compared the obtained values with the maximum permissible concentration of heavy metals in soil. For lead and arsenic, the maximum permissible concentrations were employed in accordance with the standards set forth by the Kazakhstani government [71]. For other metals, data were sourced from literature [31] or Russian GOST [72] (Table 7).

**Table 7:** Exceedance of soil maximum permissible concentration (MPC) indicators by average values of heavy metal content in solid snow sediment in Pavlodar City.

Trace element	MPC of soils	Exceeding the MPC value of the Eastern industrial zone	Exceeding the MPC value of the Northern industrial zone	Exceeding the MPC value Residential areas of the city	Exceeding the MPC value for the city as a whole
Zn	55	3.4	5.2	7.9	5.0
As	2.0	6.7	7	4.2	6.3
Cd	0.5	17.7	3.4	3.4	8.3
Pb	32	3.1	5	6.8	3.6
Cr	100	2.4	3.2	4.3	3.5
Co	5	1.8	2.4	1.8	2.0
Ni	20	1.2	1.6	1.3	1.4
Cu	33	3.2	3.9	3.9	3.7
Mo	5	0.4	0.5	0.4	0.4
V	150	0.6	0.9	0.6	0.8
Mn	700	0.9	0.9	0.9	0.9
Sr	10	25.8	26.7	27.2	26.4

Our calculations indicate that the heavy metal content of the snow in the eastern industrial zone, which is affected by aluminium production and a thermal power plant, as well as in the northern and residential zones, exceeds the maximum permissible concentrations for soils. In conclusion, the excess of maximum concentrations in the eastern zone is the lowest compared to other areas of the city. This assessment of heavy metal content in relation to the MPC is indicative. Firstly, it should be noted that the heavy metal content of solid sediment is always higher than that of soil, due to the melting, flushing, and infiltration of melt water. Secondly, the MACs are designed for agricultural lands with the objective of preventing contamination of plant products. However, this study focused on industrial lands.

The quality of the melt water was evaluated in accordance with established water quality standards. A comparison of the concentrations of highly toxic elements in the snow mass with the maximum permissible concentrations approved for surface waters indicates that the soluble forms are present in insignificant quantities. In accordance with the Unified System of Classification of Water Quality in Water Bodies, which has been adopted in the Republic of Kazakhstan, there is no evidence of an excess for any of the classes of surface water pollution.

Exceeding these thresholds can lead to both non-cancer and cancerous health effects and requires careful health risk assessment. Lead is a known neurotoxin that can cause serious health problems, particularly in children, including developmental delays and cognitive impairment [73,74]. Chronic exposure to lead can lead to a number of health problems, including high blood pressure and kidney dysfunction in adults [75].

Cadmium is another heavy metal of concern due to its toxic effects and classification as a human carcinogen [73]. It enters the human body mainly through contaminated food and water, leading to kidney damage and brittle bones [75].

High levels of zinc can cause gastrointestinal problems and interfere with the ab-sorption of other essential metals such as copper [67,76]. Arsenic is best known for its carcinogenic properties and has been linked to a variety of cancers, including skin, bladder and lung cancer [67,73].

The health effects of lead exposure from oil refining are significant. Lead is a known neurotoxin that can cause a range of health problems, particularly in children, including developmental delays and cognitive impairment [77]. Chronic ex-posure to lead and other heavy metals through contaminated water and soil can have serious long-term health consequences for communities living near oil refineries [78].

Standard health risk assessment methods used to analyse contaminated soil are not suitable for assessing snow cover. This is because direct exposure to snow is minimal. Unlike soil, snow is rarely inhaled or ingested by humans, so the main routes of exposure to contaminants are eliminated. Skin exposure to snow is also negligible, as people usu-ally wear clothing that minimises contact with precipitation.

However, children may be particularly vulnerable to contaminated snow, especially when playing outside and interacting with snow. Young children may accidentally ingest snow or inhale suspended particles of pollutants released into the air by snow evapora-tion or play. This factor is particularly relevant in areas with high levels of heavy metals in the snow cover, such as lead and cadmium, making snow cover monitoring an im-portant element of environmental monitoring.

In our study, we used an environmental risk index (Er) to assess the potential tox-icity of metals in sediments, which is particularly relevant in areas affected by industrial emissions [79,80].

Taking into account the reaction coefficient of toxicity of a single metal (Mn, Cd, Cr, V, Pb, Cu, Zn, Ni), we calculated the ecological risk index of snow pollution by heavy metals in the city as a whole, which was 192.13, which corresponds to a high potential ecological risk, if we use the data on soils [48] for interpretation. In addition, using literature data on background levels in pristine areas [31], we also calculated the ecological risk and its value was 939.5, which corresponds to a very high potential ecological risk.

### Total pollution assessment

In order to assess the overall pollution levels, pollution coefficients were calculated for each individual element. Consequently, the total pollution index across the entire city was 11.3, while for residential areas and the northern industrial zone, it was 10.2 and 10.9, respectively. It is noteworthy that the eastern industrial zone exhibited the highest index, at 18.2. Although all values indicate a low level of pollution, some authors have attributed values ranging from 16 to 24 to the general urban pollution level. Nevertheless, the low index in the total industrial area may indicate elevated levels of background heavy metal content, suggesting that the area selected for background sampling was influenced by anthropogenic factors. In accordance with the classification proposed by Sayet et al. in 1990 [81], the snow cover in the central part of Pavlodar city is categorised as exhibiting a moderately moderate level of pollution, while the northern and eastern industrial zones are characterised as areas with high levels of hazardous pollution. Nevertheless, the findings of our study do not entirely align with this classification. This discrepancy may be attributed to the differing assessment methods employed and the improved environmental conditions in Pavlodar city over the past three decades.

### Environmental policy of the Republic of Kazakhstan

The environmental policy of the Republic of Kazakhstan regarding the control of heavy metals in the environment is based on regulatory legal acts and the monitoring system implemented by RSE Kazgidromet. One of the key documents is the Heavy Metal Emissions Calculation Methodology approved in 2022, which aims to standardise and unify the process of assessing emissions to the atmosphere, soil and water bodies. This is part of a broader environmental policy regulated by the country’s Environmental Code, within the framework of obligations under international agreements such as the Stockholm Convention.

Kazakhstan carries out comprehensive environmental monitoring, including monitoring of atmospheric air, soil, surface and transboundary waters, precipitation and snow cover, and radiation exposure. The results of monitoring in the context of cities and regions are published in information bulletins of RSE “Kazgidromet”, which are posted on the official website of the company www.kazhydromet.kz.

Air monitoring is carried out at 170 fixed stations and with the use of mobile laboratories. These stations measure concentrations of pollutants, including suspended particulate matter (PM-2.5 and PM-10), sulphur dioxide, carbon dioxide, carbon monoxide, nitrogen dioxide and nitrogen oxides. Monitoring the chemical composition of the snowpack. Snowpack samples measure chemical composition including hydrocarbonates, sulphates, chlorides, calcium, sodium, potassium and magnesium ions. In addition, total mineralisation, specific electrical conductivity and snow acidity, which is usually in the slightly alkaline range, are assessed. Soil quality in residential and industrial areas is monitored for heavy metals (chromium, lead, zinc, copper, cadmium). In 2021, exceedances of the MACs for cadmium, lead, copper, zinc and chromium were observed in soils in industrially developed areas, near industrial enterprises and in the vicinity of major motorways.

As part of Kazakhstan’s environmental policy, there are regulations on maintaining a register of emissions and transfers of pollutants, which is an open electronic database on emissions and pollution levels. This register contains information on emissions of heavy metals - lead, cadmium, chromium, copper, nickel and zinc - into the atmosphere by energy, metallurgical and other enterprises, and helps to ensure transparency of data and public participation in decision-making in the field of environmental protection [33].

The current precipitation monitoring system in Kazakhstan does not include heavy metals such as lead, cadmium, copper, zinc and chromium. Although current programmes cover precipitation chemistry, including hydrocarbonates, sulphates, chlorides, as well as total salinity, acidity and electrical conductivity, heavy metal concentrations in precipitation remain outside the scope of analysis.

Based on our study, it is recommended that precipitation monitoring be extended to include the measurement of heavy metals. This will allow for a more accurate assessment of the impact of industrial and road traffic emissions on the environment, as well as improving the monitoring and decision-making system in the field of nature conservation.

Snow is an important indicator for assessing the state of air and soil pollution, as pollutants accumulated in the snow cover can reflect the extent of heavy metal emissions to the environment and their accumulation in soils. In addition, snow shows the actual accumulation of pollutants over a given period of time, excluding residual pollution such as in soils, and also takes into account the content of pollutants that can enter surface and groundwater with meltwater.

## Conclusions

The study confirmed that heavy metal pollution in the snow cover of Pavlodar is influenced by both industrial emissions and vehicular traffic. The highest concentrations of pollutants were found in industrial zones, with notable contamination also detected in residential areas, indicating the impact of transportation emissions. The spatial distribution of pollutants suggests a strong correlation between pollution sources and their dispersion patterns, which aligns with previous studies on atmospheric contamination [71,72].

Mapping analysis highlighted that cadmium, chromium, and manganese were closely linked to industrial and traffic sources. The presence of these elements in high concentrations in specific zones supports the hypothesis of localized pollution from industrial activities and transport systems. The study also showed that heavy metal levels in snow exceeded permissible concentrations in certain areas, raising concerns about potential environmental and health risks, particularly in industrial and residential zones [4,5].

The findings suggest that snow can serve as an effective environmental monitoring tool, providing valuable insights into pollution levels and their spatial distribution. The integration of snow cover analysis into existing environmental monitoring programs can improve the assessment of urban pollution impacts and inform sustainable urban planning policies. Addressing these environmental challenges requires targeted policy measures and continued research on pollution mitigation strategies [11].

Future studies should focus on long-term monitoring of snow pollution trends to evaluate the effectiveness of pollution control measures. Additionally, further investigation into the chemical forms of heavy metals in snow and their potential bioavailability could provide a more comprehensive understanding of their environmental impact.

## Supporting information

S1 Table The scale for interpreting the obtained values of the phase distribution coefficient [47]. (DOCX)

S2 Table Concentration, background concentration and concentration coefficient (Kc) of the trace elements in the solid snow sediment in Pavlodar. (DOCX)

S3 Table Comparative characteristics of heavy metals in solid snow sediment by city districts. (DOCX)

S4 Table Comparison of chemical species concentrations in snow under various conditions, all values in µg/l [11,22,23]. (DOCX)

S5 Table Comparative characteristics of heavy metals in melted water by city districts. (DOCX)
