# Supplementary material for: Assessing the spatial distribution and sources of heavy metal pollution in the snow cover: A case study from Pavlodar, Northeastern Kazakhstan
Source: PLoS One. 2025 May 12;20(5):e0322300. doi: 10.1371/journal.pone.0322300 (PMC12068655; doi:10.1371/journal.pone.0322300)
Supplement: S1 Table — (DOCX) [file pone.0322300.s001.docx]

**S1 Table.** **The scale for interpreting the obtained values of the phase distribution coefficient [1].**

| Values | Phase predominance |
| --- | --- |
| > 10 | very high predominance of the suspended form of the element |
| 5–10 | high predominance of the suspended form |
| 2–5 | moderate predominance of the suspended form |
| ≈ 1 | approximate equality of suspended and dissolved forms |
| <0.5 | predominance of the dissolved form of the element |

**References**

1. Markova YL. **Abstract of the dissertation of the Candidate of Geological Sciences.** Assessment of the impact of industry and transport on the ecosystem of the Losiny Ostrov National Park. Moscow: MSU; 2003. Russian.
