# Supplementary material for: Assessing the spatial distribution and sources of heavy metal pollution in the snow cover: A case study from Pavlodar, Northeastern Kazakhstan
Source: PLoS One. 2025 May 12;20(5):e0322300. doi: 10.1371/journal.pone.0322300 (PMC12068655; doi:10.1371/journal.pone.0322300)
Supplement: S2 Table — (DOCX) [file pone.0322300.s002.docx]

# **S2 Table. Concentration, background concentration and concentration coefficient (Kc) of the trace elements in the solid snow sediment in Pavlodar.**

| Trace element | Hazard Class | Min, mg/kg | Max, mg/kg | Mean, mg/kg | Standard Deviation | Coefficient of Variation | Background concentration | The concentration coefficient |
| --- | --- | --- | --- | --- | --- | --- | --- | --- |
| Zn | 1 | 63 | 1300 | 274.6±45* | 255 | 93 | 175 | 1.6 |
| As | 1 | 6 | 37 | 12.6±1.1 | 6.1 | 48 | 7 | 1.8 |
| Cd | 1 | 0.3 | 33 | 4.1±1.3 | 7.3 | 175 | 0.8 | 5.2 |
| Pb | 1 | 20 | 430 | 114±15 | 85 | 75 | 42.5 | 2.7 |
| Cr | 2 | 59 | 1200 | 346.9±48.4 | 273.6 | 79 | 755 | 0.5 |
| Co | 2 | 6 | 22 | 10.1±0.6 | 3.7 | 36 | 7.5 | 1.4 |
| Ni | 2 | 12 | 49 | 28.3±1.7 | 9.7 | 34 | 20.5 | 1.4 |
| Cu | 2 | 35 | 430 | 121±17.8 | 100.9 | 83 | 70 | 1.7 |
| Mo | 2 | 1 | 4.2 | 2.2±0.2 | 0.8 | 36 | 1.1 | 2 |
| V | 3 | 64 | 720 | 114±19.9 | 112.9 | 99 | 64 | 1.7 |
| Mn | 3 | 400 | 1800 | 638.1±48.9 | 276.7 | 43 | 560 | 1.13 |
| Sr | 3 | 200 | 490 | 263.7±12.2 | 68.9 | 26 | 205 | 1.3 |
| Ba | 3 | 570 | 2200 | 949.4±60.9 | 344.5 | 36 | 990 | 0.95 |

*Standard Error of the Mean
