# Supplementary material for: Assessing the spatial distribution and sources of heavy metal pollution in the snow cover: A case study from Pavlodar, Northeastern Kazakhstan
Source: PLoS One. 2025 May 12;20(5):e0322300. doi: 10.1371/journal.pone.0322300 (PMC12068655; doi:10.1371/journal.pone.0322300)
Supplement: S3 Table — (DOCX) [file pone.0322300.s003.docx]

**S3 Table. Comparative characteristics of heavy metals in solid snow sediment by city districts.**

| Area | The decreasing sequence  by average content | The decreasing sequence  by coefficient of variation | The decreasing sequence  by concentration coefficient |
| --- | --- | --- | --- |
| Northern industrial zone | Ba_1076,4_>Mn_638,1_>Cr_316,6_>Zn_286,2_>Sr_267,1_>Pb_158,5_>V_141,7_>Cu_127,2_>Ni_32,9_>As_14_>Co_11,8_>Mo_2,5_>Cd_1,67_ | V_119_>Cr_105_>Cu_87_>Cd_82_>Zn_72_>Pb_64_>Mn_54_>As_54_>Ba_42_>Mo_40_>Co_37_>Sr_31_>Ni_30_ | Pb_3,7_>Mo_2,3_>V_2,2_>Cd_2,09_>As_2_>Cu_1,8_>Zn_1,6_>Ni_1,6_>Co_1,57_>Sr_1,3_>Mn_1,14_>Ba_1,08_>Cr_0,4_ |
| Eastern industrial zone | Ba_777,5_>Mn_658,3_>Cr_259,1_>Sr_258,3_>Zn_187,5_>Cu_107,5_>Pb_98,4_>V_97,9_>Ni_24,8_>As_13,4_>Co_9,08_>Cd_8,84_>Mo_2,03_ | Cd_118_>Cu_92_>Zn_78_>Pb_53_>Mn_39_>As_34_>Cr_33_>Ni_33_>Co_29_>Sr_25_>Ba_25_>Mo_23_>V_21_ | Cd_11,1_>Pb_2,3_>As_1,9_>Mo_1,8_>Cu_1,5_>V_1,5_>Sr_1,3_>Ni_1,2_>Co_1,2_>Mn_1,2_>Zn_1,1_>Ba_0,8_>Cr_0,3_ |
| Residential area of ​​the city | Ba_886,6_>Mn_608,3_>Zn_436,6_>Cr_431,6_>Sr_271,6_>Pb_218,16_>Cu_128,3_>V_84,1_>Ni_26,5_>Co_9,1_>As_8,3_>Mo_1,9_>Cd_1,68_ | Pb_175_>Zn_101_>Cu_75_>Cr_59_>Cd_41_>As_32_>Ni_29_>Co_23_>Ba_22_>Mo_22_>Mn_19_>Sr_15_>V_11_ | Pb_5,1_>Zn_2.5_>Cd_2,1_>Cu_1,8_>Mo_1.7_>Sr_1,3_>V_1,3_>Ni_1,3_>Co_1,2_>As_1,2_>Mn_1,1_>Ba_0,9_>Cr_0,6_ |
| City average | Ba_949,4_>Mn_638,1_>Cr_346,9_>Zn_274,6_>Sr_263,7_>Cu_121_>V_114_>Pb_114_>Ni_28,3_>As_12,6_>Co_10,1_>Cd_4,1_>Mo_2,2._ | Cd_175_>V_99_>Zn_92_>Cu_83м_>Cr_78_>Pb_74_>As_48_>Mn_43_>Ba_36_>Co_36_>Mo_36_>Ni_34_>Sr_26_ | Cd_5,2_>Pb_2,7_>Mo_2_>As_1,8_>Cu_1,7_>V_1,7_>Zn_1,6_>Co_1,4_>Ni_1,4_>Sr_1,3_>Mn_1,13_>Ba_0,98_>Cr_0,5_ |
