# Supplementary material for: Assessing the spatial distribution and sources of heavy metal pollution in the snow cover: A case study from Pavlodar, Northeastern Kazakhstan
Source: PLoS One. 2025 May 12;20(5):e0322300. doi: 10.1371/journal.pone.0322300 (PMC12068655; doi:10.1371/journal.pone.0322300)
Supplement: S4 Table — (DOCX) [file pone.0322300.s004.docx]

**S4 Table.** **Comparison of chemical species concentrations in snow under various conditions, all values in µg/l.**

| Trace element | This study | National park, Poland [1] | Rock mining, Poland [2, 3] | Metallurgical production, Poland [4] | Urban, Poland [5] | Urban, Russia [6] | Urban (impact of transport), Russia [7] | Gobi Desert, agricultural land, China [8] | Wetlands, China [9] | Urban, China [10] |
| --- | --- | --- | --- | --- | --- | --- | --- | --- | --- | --- |
| V | 5.1 |  |  |  |  | 3.3 | 12 | 0.7 |  |  |
| Cr | 1.9 | 0.3 |  | 0.6 | 0.4 | 0.4 |  | 0.9 | 11.7 | 41.1 |
| Mn | 26 |  |  |  |  |  | 55 | 22 |  |  |
| Cu | 4 |  |  |  | 2 | 2.3 | 17 | 1.3 | 6.2 | 56.9 |
| Zn | 58.6 | 4.9 | 48.8 | 57.1 | 13.2 | 18 | 33 | 23 | 103.5 | 62 |
| As | 3 |  |  |  | 0.7 |  | 0.27 | 3 |  |  |
| Pb | 4.7 | 0.5 | 7.7 |  | 4.9 | 0.5 | 5 | 2.5 | 13.1 | 42.7 |

**References**

1. Kozłowski R., Szwed M., Przybylska J. Physico-chemical properties of snow in the city of Kielce in January 2016. Proc. ECOpole. 2017;11:185–191. doi:10.2429/proc.ecopole.2017.11.1.22.
2. Kozłowski R., Jarzyna K., Jóźwiak M., Szwed M. Influence of cement-lime industry on the physico-chemical and chemical properties of snow cover in a “Białe Zagłębie” region in February 2012. Monit. Sr. Przyr. 2012;13:71–80.
3. Kozłowski R., Szwed M. Heavy metals content in the snow cover in the Holy Cross Mountains. Monit. Sr. Przyr. 2016;18:61–69.
4. Jarzyna K., Kozłowski R., Szwed M. Chemical properties of snow cover as an impact indicator for local air pollution sources. Infrastruktura i Ekologia Terenów Wiejskich. 2017;1591-1607.
5. Siudek P., Frankowski M., Siepak J. Trace element distribution in the snow cover from an urban area in central Poland. Environ Monit Assess. 2019;187:225. doi:10.1007/s10661-019-7298-0.
6. Kondrat’ev I.I., Mukha D.E., Boldeskul A.G., Yurchenko S.G., Lutsenko T.N. Chemical composition of precipitation and snow cover in the Primorsky krai. Russ. Meteorol. Hydrol. 2014;42:64–70. doi:10.3103/S1068373914010097.
7. Vlasov D., Vasil’chuk J., Kosheleva N., Kasimov N. Dissolved and suspended forms of metals and metalloids in snow cover of megacity: partitioning and deposition rates in Western Moscow. Atmosphere. 2020;11:907. doi:10.3390/atmos11090907.
8. Wang X., Pu W., Zhang X., Ren Y., Huang J. Water-soluble ions and trace elements in surface snow and their potential source regions across northeastern China. Atmospheric Environment. 2015;114:57–65. doi:10.1016/j.atmosenv.2015.05.049.
9. Zhang F, Meng B, Gao S, Hough R, Hu P, Zhang Z, Yu S, Li K, Liu Z, Cui S. Levels, inventory, and risk assessment of heavy metals in wetland ecosystem, Northeast China: implications for snow cover monitoring. Water. 2021;13(16):2161. doi:10.3390/w13162161.
10. Cui S., Li K.Y., Fu Q., Liu D., Dong W.C., Li T.X. Pollution characteristics and inventory estimation of heavy metals in snow cover in Harbin. J. Basic Sci. Eng. 2019;27:1248–1257. doi:10.3969/j.issn.1005-0930.2019.06.014.
