# Supplementary material for: Assessing the spatial distribution and sources of heavy metal pollution in the snow cover: A case study from Pavlodar, Northeastern Kazakhstan
Source: PLoS One. 2025 May 12;20(5):e0322300. doi: 10.1371/journal.pone.0322300 (PMC12068655; doi:10.1371/journal.pone.0322300)
Supplement: S5 Table — (DOCX) [file pone.0322300.s005.docx]

**S5 Table.** **Comparative characteristics of heavy metals in melted water by city districts.**

| Area | The decreasing sequence  by average content | The decreasing sequence  by coefficient of variation | The decreasing sequence  by concentration coefficient |
| --- | --- | --- | --- |
| Northern industrial zone | Sr_44,7_>Zn_39,6_>Mn_24,9_>Ba_23,3_>V_4,8_>Pb_3,9_>Cu_3,4_>As_3,3_>Cr_1,4_ | Zn_156_>V_138_>As_134_>Sr_105_>Ba_90_>Cu_61_>Cr_61_>Mn_50_>Pb_39_ | Cr_3,4_>Sr_2,9_>Zn_2,7_>Cu_2,3_>V_2,1_>Pb_1,6_>Mn_1,6_>As_1,5_>Ba_1,2_ |
| Eastern industrial zone | Mn_28,3_>Sr_27,8_>Ba_19,1_>Zn_13,2_>V_6,7_>Pb_5,1_>Cu_2,9_>As_2,3_>Cr_1,4_ | Zn_98_>Cr_80_>Mn_65_>Ba_58_>As_51_> V_45_>Cu_42_>Pb_37_>Sr_23_ | Cr_3,5_>V3>Pb_2,2_>Cu_1,9_>Mn_1,8_>Sr_1,8_As_1,1_>Ba1>Zn_0,9_ |
| Residential area of the city | Zn_32,6_>Sr_31_>Mn_28,7_>Ba_23,3_> Pb_6,7_>V_3,3_>Cu_2,7_>As_2,3_>Cr_1._ | Cu_145_>Cr_82_>Pb_62_>V_56_>Mn_53_> Ba_46_>Sr_41_>Zn_37_>As_28_ | Pb_2,8_>Cr_2,5_>Zn_2,2_>Sr_2_>Cu_1,8_>Mn_1,8_>V_1,4_>Ba_1,2_>As_1,1_ |
| City average | Zn_58,6_>Sr_34,8_>Mn_26_>Ba_21,9_>V_5,1_>Pb_4,7_>Cu_4_>As_3_>Cr_1,9_ | Zn_146_>Mo_100_>As_99_>V_96_>Sr_92_> Ba_71_>Mn_58_>Cu_54_>Pb_45_>Cr_44_ | Cr_4,4_>Mo_4,3_>V_2,3_>Sr_2,2_ >  Cu_2_ >Zn_2_ >Pb_1,9_>Mn_1,6_> As_1,4_>Ba_1,1_ |
